# War and Eugenics: A Paroxysm of Paradox

**Published:** 1917-04-21

**Authors:** 


### WAR AND EUGENICS.

### A Paroxysm of Paradox.

By CADUCEUS.

Eugenists seem at first sight to have a strong case against war. Physically selected men of the age when the body is most vigorous are taken from their homes and exposed to risk, great and prolonged, of death or disablement, while to the old and feeble and undersized and diseased is largely left the *rôle* of becoming fathers of the succeeding generation. So much is unanswerable, and the proposition that war is anti-eugenic had for some time been adopted, and indeed made use of as an argument by pacifists, when, soon after the outbreak of the present great conflict, some attempt was made to put a better face upon matters. It was recalled that soldiers nowadays are often very young, and that Aristotle had put the age limit of desirable fatherhood as high as fifty-five years; also that unfitness for military service is sometimes not incompatible with good, even with exceptional, physical powers. These counter-pleas did not, however, bring conviction; the encouragement of war-marriages, doubtless due to desire of quality as well as to the wish for replenishment, proved as much; and it would now be best, we think, to admit that, in addition to its manifold other evils, war has the effect of damaging national physique, not only in the present but also, to an indeterminate extent, in the future.

Yet, for all that, war may in part be eugenic. For, while not, to be sure, the original founders, still many of the popularisers, of eugenics, especially those who from wilfulness or from the momentum of their previous propagandism confused it with puericulture, looked on the subject too narrowly, made it concerned only with bodily traits, not with mental ones as well. How to breed brains, talent, perhaps genius, is surely as important a study as how to breed brawn and stature and good eyesight. If war certainly stands in the way of the latter object, at least there is still to be proved that it defeats the former one too. And indeed the far from unlikely thing is that it may actually further it: that the two ends are opposed, that breeding from the physically superior means breeding from the mentally inferior, and *vice versa*, that the slaughter of war may possibly bequeath to a nation a population more intellectual than its progenitors. For such verification of this theory as can be attempted one must of course look to history, a vast task of which only a tiny fraction can be here attempted, and that in the merest outline. Since, at any rate, the close of the mediæval epoch, Great Britain, because of its geographical position, has been on the whole much less war-harried than the Continental peoples. Napoleon by his pursuit of glory lowered the average height of Frenchmen an inch, or so it is said. At the time of his fall, writes M. Anatole France with a satirist's licence, there were left in France "none but the hunchbacks and cripples from whom we are descended." But at the same time the speculation may in fairness be hazarded that the great soldier had similarly something to do with the position of Paris, at the end of the century whose beginning he troubled, as the artistic capital of the world. This speculation, indeed, is but the converse of Galton's—and that was well received—that the backwardness of Roman Catholic countries, and especially the decline of Spain, may in part be laid to the charge of the institution of clerical celibacy, whereby the finer-minded of the community were sterilised for many generations continuously. At least this parallel may be claimed if one admission is made—namely, that mental distinction is often accompanied by physical defect. But most people except purblind optimists will allow enough truth to Lombroso's doctrine for that.

The last threads of our argument are not comforting to patriotic sentiment, it must be owned, although perhaps one had better say rather to superficially patriotic sentiment. During the last half-dozen generations, Englishmen and Americans have not been fighting nearly so often or so desperately as have their contemporaries of the Continent of Europe. And the Briton or the American of the present day is noticeably less given to the things of the mind than the Continental is. Not that it could be maintained for a moment that this consequent has that single antecedent, for there are many others the discussion of which lies outside the province of a medical journal. But the coincidence is all the same suggestive. In England or the United States one does not see city streets named after scientists, as in the case of the Place Raspail at Lyons or Paul Ehrlich Strasse in Frankfort. The drama, at any rate, is on a lower level, music an exotic, athletics a fetish. The Anglo-Saxon, says M. Bourget, shows at his best on the polo ground or the cricket field. In London a world poet, Milton, has only the third of a pedestal. Yet the slaughter of six million Europeans may be attended by this gleam of consolation, the hope and expectation, namely, of a higher average standard of intelligence in those who come after them.

[We print these views of an old and valued contributor, as received; but we think there will be many who will dissent from his premises and will therefore not admit his conclusions. To take one point only—the main pivot of his whole argument; this is the statement that mental distinction is often accompanied by physical defect. As it stands, no one can deny that the statement is true, provided one is allowed to give one's own interpretation to the word "often." But if the author means, as he appears to mean, that the general level of mental distinction amongst the physically defective is higher than amongst the physically elect, then we cannot accept his view, nor the arguments he founds upon it.—Ed. The Hospital.]

